# Plastid Phylogenomic Analyses Reveal a Cryptic Species of *Ligusticopsis* (Apiaceae, Angiosperms)

**DOI:** 10.3390/ijms24087419

**Published:** 2023-04-18

**Authors:** Rongming Tian, Xueyimu Aou, Boni Song, Zixuan Li, Xingjin He, Songdong Zhou

**Affiliations:** Key Laboratory of Bio-Resource and Eco-Environment of Ministry of Education, College of Life Sciences, Sichuan University, Chengdu 610065, China

**Keywords:** *Ligusticopsis*, cryptic species, morphology, plastome, phylogenetic analysis

## Abstract

*Ligusticopsis litangensis* is identified and described as a cryptic species from Sichuan Province, China. Although the distribution of this cryptic species overlaps with that of *Ligusticopsis capillacea* and *Ligusticopsis dielsiana*, the morphological boundaries between them are explicit and have obviously distinguishable characters. The main distinguishing features of the cryptic species are as follows: long conical multi-branched roots, very short pedicels in compound umbels, unequal rays, oblong-globose fruits, 1–2 vittae per furrow and 3–4 vittae on the commissure. The above-mentioned features differ somewhat from other species within the genus *Ligusticopsis*, but generally coincide with the morphological boundaries defined for the genus *Ligusticopsis*. To determine the taxonomic position of *L. litangensis*, we sequenced and assembled the plastomes of *L. litangensis* and compared them with the plastomes of 11 other species of the genus *Ligusticopsis*. Notably, both phylogenetic analyses based on ITS sequences and the complete chloroplast genome robustly supported that three accessions of *L. litangensis* are monophyletic clade and then nested in *Ligusticopsis* genus. Moreover, the plastid genomes of 12 *Ligusticopsis* species, including the new species, were highly conserved in terms of gene order, gene content, codon bias, IR boundaries and SSR content. Overall, the integration of morphological, comparative genomic and phylogenetic evidence indicates that *Ligusticopsis litangensis* actually represents a new species.

## 1. Introduction

*Ligusticopsis* Leute is a genus of Apioideae within the Apiacae family, which was established by Leute in 1969 with the type species *Ligusticopsis rechingeriana* Leute [1], containing 14 species. Subsequently, Pimenov recognized 18 species of *Ligusticopsis* in China based on morphological observation and specimen examination [2]. However, this genus has not been commonly accepted and its independent status has been controversial due to the blurred morphological delimitation with the genus *Ligusticum*. For instance, some scholars have supported the merging of *Ligusticopsis* into *Ligusticum* solely by morphological researches of pollen, fruit and leaf epidermis [3,4]. Nevertheless, Li et al. [5] recently conducted a comprehensive research of the genus *Ligusticopsis* and clearly demonstrated that *Ligusticopsis* is a separate genus based on phylogenetic reconstruction, plastid comparative genomic studies and morphological approaches. They identified nine “true *Ligusticopsis* species” and redefined the morphological delimitations of *Ligusticopsis*, including fibrous remnant sheaths at the stem base, nearly equal umbrella length, pinnate bracts, bracteoles longer than the umbrella length and rarely undivided, well-developed calyx teeth, strongly compressed back of mericarps, dorsal and intermediate ribs filamentous to keel convex, winged lateral ribs and multiple long vittae in each furrow and commissure [5]. Likewise, the results of the plastid phylogenomics of the genus *Ligusticum* by Ren et al. [6] further support the conclusion that the genus *Ligusticopsis* has an independent phylogenetic status. Additionally, the relevant molecular phylogenetic research indicates that *Ligusticopsis* is positioned in the Selineae of the Apioidae [7]. In conclusion, the above-mentioned studies associated with the genus *Ligusticopsis* provide a powerful foundation for research on this new species.

The Hengduan Mountain region (HDM) in southwest China is one of the 34 biodiversity hotspots on Earth and has the most flora diverse in the North temperate zone [8]. Litang County is located in the middle of Hengduan Mountain Range, with an average elevation of more than 4000 m. The region has experienced complex geographical and climatic changes, leading to diverse species and unique floras [9].

**Figure 1:**
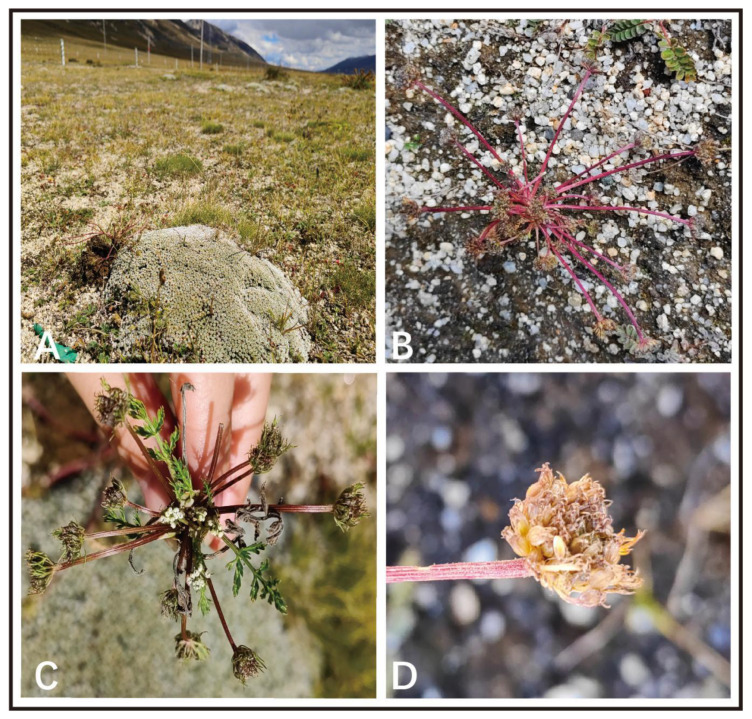
Habitat and morphological features of *Ligusticopsis litangensis* (**A**) habitat; (**B**,**C**) plant; (**D**) fruit.

During a botanical expedition related to the Apiaceae species in Litang County in 2022, a distinctive Apiaceae species with short peduncles, unequal rays and pinnately divided bracteoles was collected (Figure 1). We discovered a species that is incongruent with other known analogous species and investigated this taxon in detail in terms of morphology and molecular phylogeny. Consulting a large number of specimens and making detailed field investigations, we found this species is close to *Ligusticopsis* species but is distinctly different from all previously published *Ligusticopsis* members, which was also supported by molecular phylogenetic analyses. Hence, integrating the morphological, genomic and phylogenetic evidence, we found that the species actually represents a new species.

## 2. Results

### 2.1. Morphological Analysis

**Figure 2:**
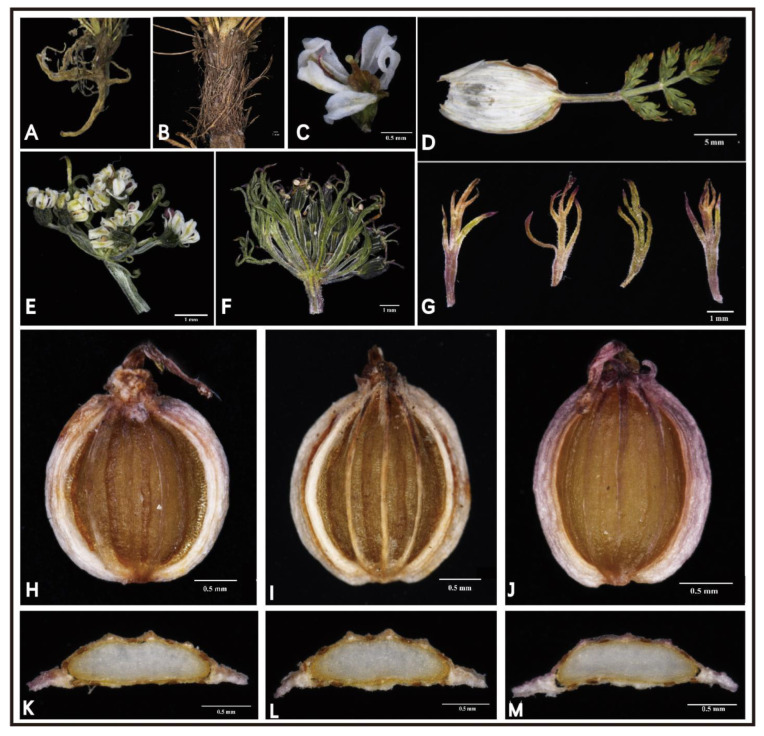
Morphology of *Ligusticopsis litangensis* (**A**) root; (**B**) stem (base); (**C**) flower; (**D**) leaf; (**E**) inflorescence; (**F**) infructescence; (**G**) bracteoles; (**H**–**J**) oblong-globose fruit; (**K**–**M**) mericarp transverse section.

Several specimens of *L. litangensis* were collected from Litang County, Sichuan Province, growing in the alpine meadows at an elevation of 4100–4300 m. We performed detailed macroscopic and micromorphological anatomical characterization of this unknown species in the laboratory. Hence, we compared and analyzed the morphological characteristics of *L. litangensis* and related species (*L. capillacea*, *L. hispida*, *L. rechingeriana*) (Table S1), such as the stem base covered with fibrous remnant sheaths, the clearly developed calyx teeth and the fruit oblong-ovoid dorsoventrally compressed with the enlarged and winglike lateral ribs of *L. litangensis*, and found that they are shared with other species of *Ligusticopsis*. However, the significant characteristics of this new species (extremely conspicuous single umbels, unequal length umbrellas and a small number of commissural vittae) obviously differ from those of the other described *Ligusticopsis* members. During our field investigations, when we first spotted this unknown species, at first glance some of its external morphological features resembled those of *Cortia depressa*. However, after careful indoor morphological anatomical examination, we identified numerous very conspicuous and distinguishable morphological features between these two species, particularly mirrored in the mericarp structure, which is an essential discriminating feature in Apiaceae (Figure 2). For example, *L. litangensis* and *C. depressa* were dominated by single umbels, but *L. litangensis* was distinguished from *C. depressa* by the fact that the bracteoles were longer than the umbellules and the dorsal and intermediate ribs were keeled. Consequently, we are able to conclude that the morphological characteristics of *L. litangensis* coincide with the morphological boundaries of the genus *Ligusticopsis*, such as the root neck densely covered with a fibrous withered leaf sheaths, pinnate bracts, calyx teeth developed, strongly compressed mericarps, keeled dorsal and middle ribs, winged lateral ribs, 1–2 vittae per furrow and 3–4 vittae on the commissure.

### 2.2. Comparative Plastome Analyses

**Figure 3:**
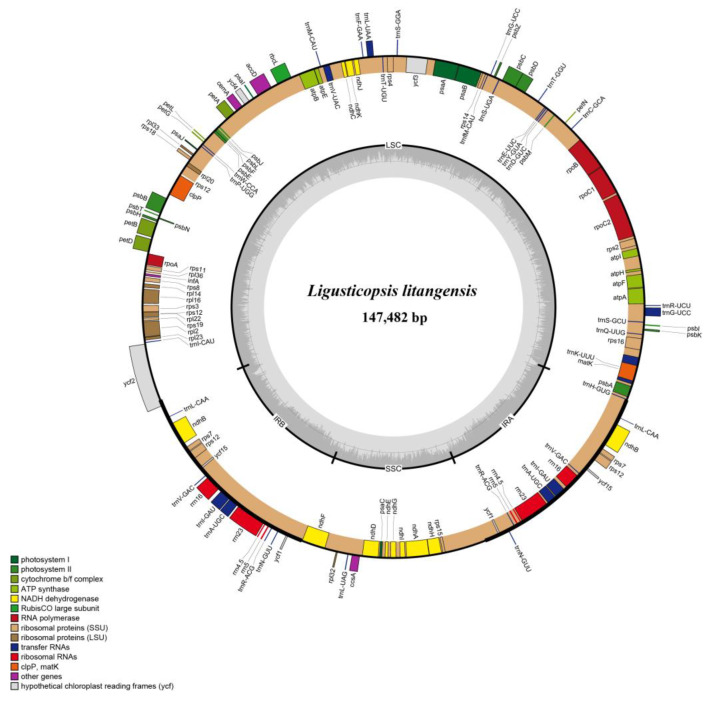
Gene map of the *Ligusticopsis litangensis* chloroplast genome.

The total length of 12 plastomes of newly sequenced *L. litangensis* and related groups downloaded online ranged from 147,482 bp to 148,633 bp (Table 1). Each of the twelve plastomes exhibited the typical quadripartite structure consisting of a pair of IR regions (19,127–19,529 bp) separated by LSC regions (91,559–92,305 bp) and SSC regions (17,503–17,669 bp). There was little difference in the total GC content of the twelve plastomes and the GC content of the LSC, SSC and IR regions. However, the IR region had a higher GC content (43.6–44.1%) than the other two regions (LSC, 35.9–36.0%; SSC, 30.9–31.0%). The typical quadripartite structure of the *L. litangensis* genome is shown in Figure 3. Under the unified parameter setting and annotation standard, 129 genes were annotated in the whole plastome of *L. litangensis*, which included 85 protein-coding genes (PCGs), 36 transfer RNA genes (tRNAs), 8 ribosomal RNA genes (Ribosomal RNA genes), rRNAs) and 2 pseudogenes.

**Figure 4:**
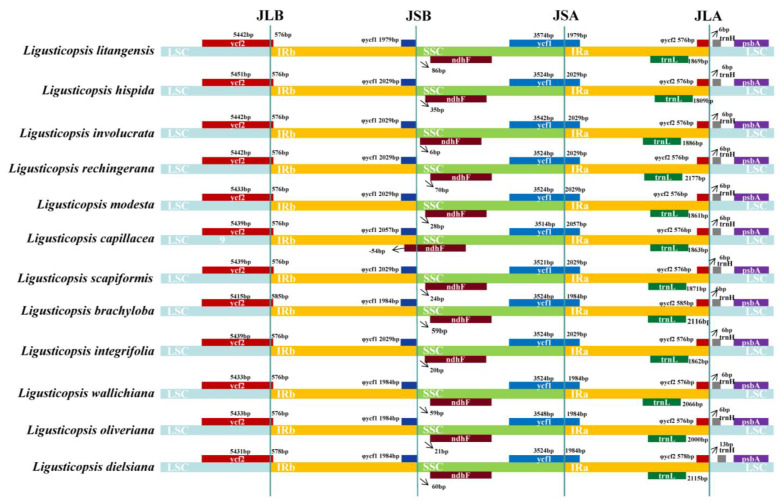
Comparison of LSC, SSC and IR boundary regions in 12 plastomes. The different boxes represent the location of the gene. JLA: the junction of the LSC and IRa. JLB: indicates the junction of the LSC and IRb. JSA: the junction of SSC and IRa. JSB: the junction of SSC and IRb.

The IR boundary map was generated by comparing the plastid genomes of 12 *Ligusticopsis* species, including the new species (Figure 4). The graphic visualizes the gene distribution in the sequence boundary region as well as the expansion and contraction of the boundary, revealing that the plastome structure and sequence boundary gene distribution are conserved and similar among these 12 *Ligusticopsis* species. Specifically, we detected comparable structures in the JLB and JLA lines of the 12 plastid genomes. For instance, the trnH gene of all 12 plastomes are situated on the right side of the JLA line, and the distance from the trnH gene to the JLA line is consistent for the new species and the other 10 plastids all at 6 bp, except for *L. dielsiana* (13 bp). Furthermore, the base number extending from the LSC to the IRb region of the ycf2 gene varied particularly insignificantly in the 12 plastomes, all ranging from 576–585 bp. Meanwhile, the base distance of the trnL gene to the JLA line varied only within a small range of divergence (1809–2177 bp). It was evident that the ndhF gene of the plastomes of the new species and 10 other species, except *L. capillacea*, was completely encompassed in the SSC region and the distance of the ndhF gene from the JSB line was minimal (6–86 bp).

**Figure 5:**
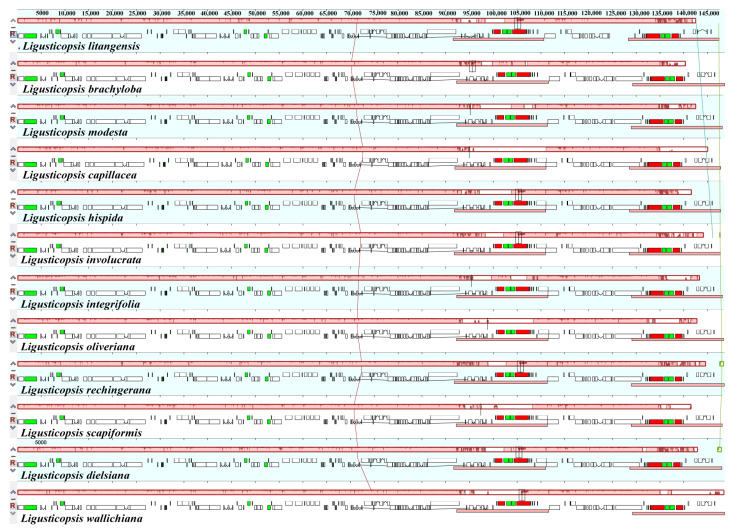
Alignment of the twelve *Ligusticopsis* plastomes (mauve graphs). Local collinear blocks within each alignment are represented by blocks of the same color connected with lines.

**Figure 6:**
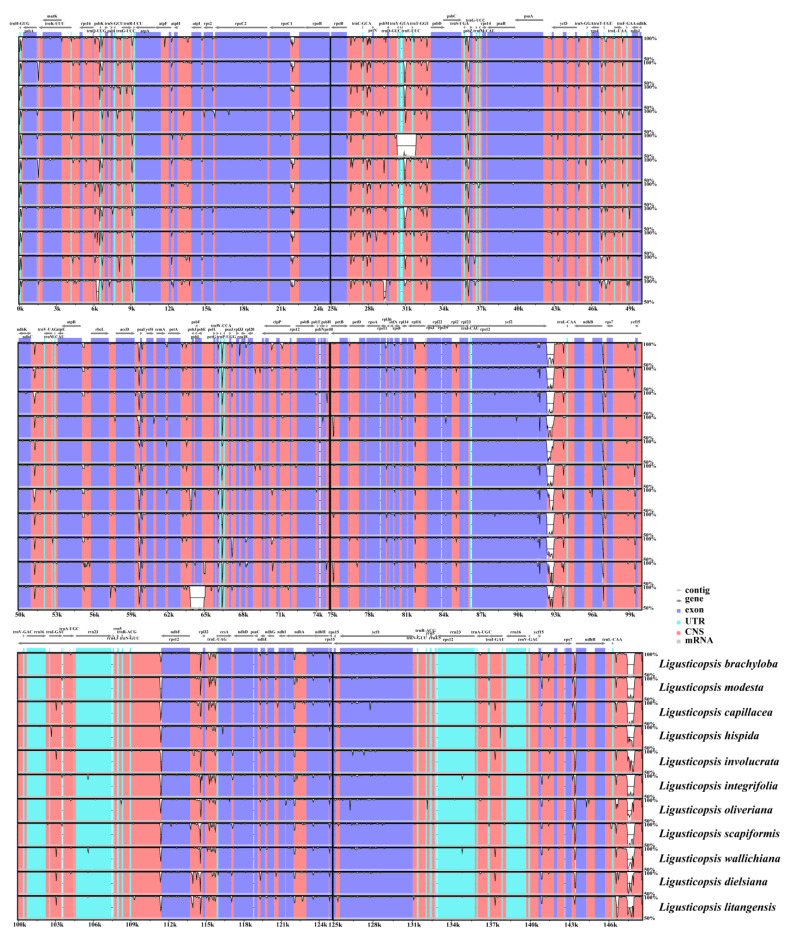
mVISTA visualization of alignment for 12 plastomes. *Ligusticopsis rechingeriana* is the reference. Blue and pink represent coding and non-coding regions, respectively. The Latin name in red represents the new species.

The mauve visualization graphs indicated that the gene arrangement in the 12 plastomes was highly conserved and no significant gene rearrangements or losses were detected (Figure 5). Using the mVISTA program, we analyzed the sequence diversity of plastomes of 12 *Ligusticopsis* species. The results demonstrated that these 12 taxa were highly conserved and the coding regions tended to be more conserved than the non-coding regions. (Figure 6). Moreover, the partial gene regions (*trnH-psbA*, *ycf1*, *ycf2*, *rpoC2*, *rpl32*, *ndhF*) exhibited a highly similar degree of differentiation. The above comparative genomic analysis showed that the plastome structure of *L. litangensis* was similar to that of other *Ligusticopsis* species, indicating the plastome structure of *Ligusticopsis* species was highly conserved.

### 2.3. Codon Usage Analyses

**Figure 7:**
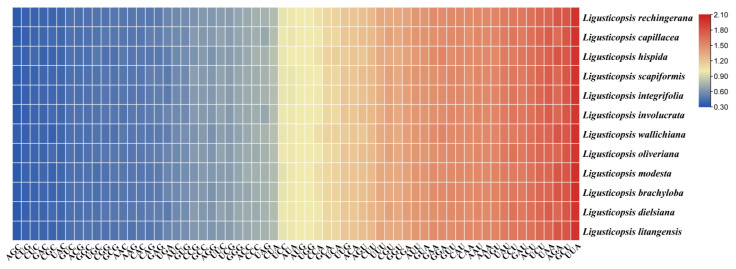
The RSCU values of all concatenated protein-coding genes for twelve plastomes. Color key: the red values mean higher RSCU values and the blue values mean lower RSCU values.

We extracted and linked 53 protein-coding genes from each species to calculate the codon usage frequency of 12 plastomes. The RSCU value is a measure of synonymous codons usage bias in a gene coding sequence, if the RSCU value of a codon is greater than 1.0, it is preferable to use the codon and vice versa. The heatmap shows the codon usage bias is similar and conserved among the 12 species of the genus *Ligusticopsis*, including the new species. These protein sequences encode 19,909–22,622 codons. Of these, the codon encoding leucine (Leu) has the most protein sequences, while the codon encoding cysteine (Cys) has the least protein sequences (Table S2). In addition, the heatmap shows that the relative synonymous codon usage (RSCU) values of all codons are between 0.34 and 2.00, and about 30 codons have RSCU values greater than 1 (Figure 7). The UAA codon had the highest value in the *L. brachyloba* plastome (RSCU = 2.0), while the AGC codon had the lowest value in *L. capillacea*, *L. involucrata* and *L. dielsiana* plastomes (RSCU = 0.34). Furthermore, all the amino acids in these 12 plastid sequences except methionine (Met) and tryptophan (Trp) were encoded by two or more codons, indicating a codon bias. Among the three termination codons (TAA, TAG, TGA), the plastomes of these 12 species had the highest RSCU values for the stop codon TAA, all of which ranged from 1.70 to 1.75.

### 2.4. Simple Sequence Repeats Analyses (SSRs)

**Figure 8:**
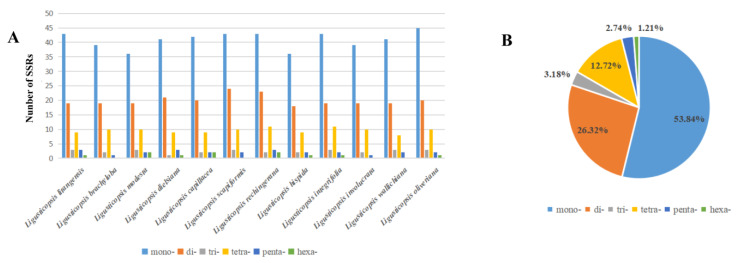
Analyses of simple sequence repeats (SSRs) in twelve plastomes. (**A**) Numbers of different repeat types; (**B**) the proportion of different SSR types in total.

We discovered the total number of SSRs varied from 68 to 84 in the 12 plastomes (Table S3). These SSR sequences were divided into six types, and the most common sequence was the single nucleotide repeat (53.84%). It was followed by the dinucleotide repeat (26.32%), tetraconucleotide repeat (12.72%), trinucleotide repeat (3.18%), pentaucleotide repeat (2.74%) and trinucleotide repeat (1.21%) (Figure 8B). Only five types of SSR (lack of hexa-) were detected in the whole genome of plastomes of *L. brachyloba*, *L. scapiformis*, *L. involucrata* and *L. wallichiana* and six types of SSR could be detected in other species of *Ligusticopsis* (Figure 8A).

### 2.5. Phylogenetic Analysis

**Figure 9:**
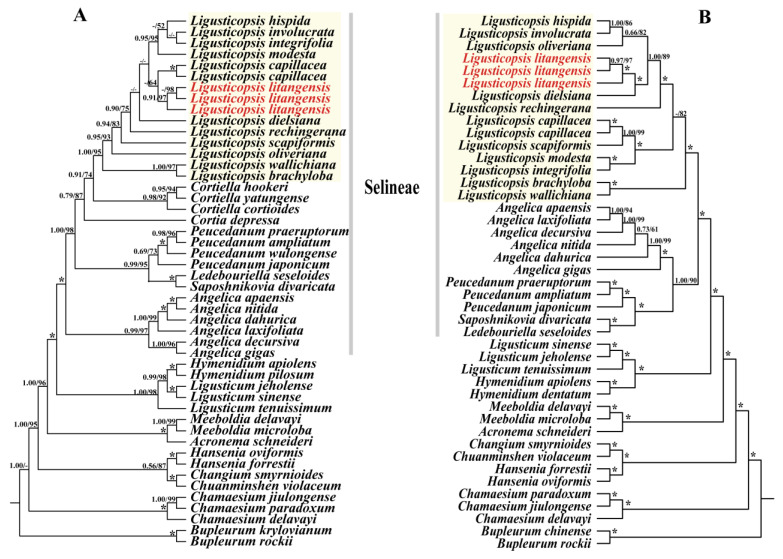
Phylogenetic relationships of *Ligusticopsis* and its related genus inferred from (**A**) ITS and (**B**) CDS based on ML and BI methods. ML BS (bootstrap) and BI PP (posterior probability) values are shown above the branches. The short line denotes values <50%. * = maximum support in both analyses. Red markers represent new species, and yellow markers represent branch of the genus *Ligusticopsis*.

We used 41 ITS sequences and 40 plastomes sequences of Apioideae for the phylogenetic analysis (Table S4). The tree topologies obtained from the ML and BI analyses based on the ITS data and plastid genome data are presented in Figure 9. It is obvious that the topologies obtained from both the ITS and plastid sequences clearly demonstrate that the 12 *Ligusticopsis* species are clustered into a stable and robust monophyletic clade located within the Selineae (ML/BS ≥ 95, BI/PP ≥ 1.00). Additionally, although the topologies between ITS sequence and plastid genome sequence were slightly different, both robustly supported that three individual sequences of *L. litangensis* are monophyletic clade and then nested in the *Ligusticopsis* genus (ML/BS ≥ 95, BI/PP ≥ 0.95).

## 3. Discussion

### 3.1. Plastome Characteristic

We report the newly sequenced and assembled complete plastomes of *L. litangensis* and compare them with 11 other species of the genus *Ligusticopsis*. The results revealed that all 12 plastomes exhibited the typical quadripartite structure containing a large single-copy region (LSC), a small single-copy region (SSC) and two inverted repeat sequence regions (IR) separating the SSC from the LSC [10,11,12,13]. Additionally, all plastomes were similar and conserved in genome size, gene order and GC content. These circumstances are more common in other genera of the family Apiaceae [14,15,16,17], which may be related to stable plastid function. Meanwhile, we also evaluated the SSRs of the plastid genomes of 12 *Ligusticopsis* species. SSRs are usually small tandem mononucleotide repeats, showing differences in the number of intraspecial repeats [18,19]. These sites are often used to develop molecular markers due to their high degree of variability [20]. For example, a hexanucleotide simple sequence repeat (ATATAC) was found in plastomes of *L. rechingerana*, but not in other plastomes, which can be used as a specific molecular marker to identify *L. rechingerana*. A total of (68–84) SSRs were obtained in this study. Most of these SSRs were mononucleotides and dinucleotides, which were consistent in number with the results of other Apiaceae [21]. The fewest SSRs were found in the plastome of *L. hispida*, which may be due to the small number of single nucleotides and the short LSC region. The research on SSRs can provide evidence for the population genetics of this genus. Consequently, these outcomes indicate that the plastid characteristics of *L. litangensis* are almost identical to other species of the genus *Ligusticopsis* and generally endorse *L. litangensis* as a new species of the genus *Ligusticopsis.*

### 3.2. Comparison of Ligusticopsis Plastomes

IR contraction and expansion are the most common causes of plastome size variation, which are very common in angiosperm plastomes [22,23,24,25]. Here, we compare the IR/SC boundaries and find that IRa/SSC/IRb/LSC overlap and the surrounding genes are identical. Hence, it can be clearly concluded that the plastid structure and sequence boundary gene distribution are conserved and similar among these 12 *Ligusticopsis* species. In terms of the degree of sequence divergence, the plastids of the new species and other species of the genus *Ligusticopsis* showed a consistent degree of sequence divergence, and the IR region is more conserved, with the most substitutions occurring in the SSC and LSC regions.

Codon bias is related to carrying genetic information and proteins with biochemical functions. The analysis of codon bias in different species may contribute to the exploration of the phylogenetic relationships between them [22,23,24]. Relative synonymous codon usage (RSCU) is a method to measure the usage bias of a synonymous codon in coding sequences. When the RSCU value is less than 1, it means no preference, while when the RSCU value is greater than 1 means that the codon is preferred. Figure 7 shows that 30 codons have RSCU values greater than 1.00, and the codons of AUG and UGG are unbiased (RSCU = 1.00). Similar relative synonymous codon usage (RSCU) values indicated that the plastomes of *L. litangensis* and other species of the genus *Ligusticopsis* have a similar codon bias, further emphasizing the validity of the status of *L. litangensis* as a new species of the genus *Ligusticopsis*. Certainly, these findings on the codon bias assist us in obtaining a deeper insight into the evolutionary process and gene expression of *Ligusticopsis*.

In conclusion, the above comparative genomic analysis showed that the plastome structure of *L. litangensis* was analogous to that of other *Ligusticopsis* species, further supporting that *L. litangensis* belongs to the genus *Ligusticopsis*.

### 3.3. Phylogenetic Analysis

Plastome is one of the three genetic systems of plants. Although the gene content and gene order of the plastid genome are usually highly conserved, it exhibits a high degree of variable characteristics. Therefore, an increasing number of researchers are utilizing plastomes for phylogenetic and comparative genomic studies [26,27,28,29,30,31,32], which help resolve many complex phylogenetic taxonomic problems.

In our research, a robust phylogenetic framework was constructed based on ITS and the plastid data to decipher the phylogenetic position of *L. litangensis*. The tree topologies obtained from ML and BI analyses based on the ITS data and plastome data firmly supported that three individuals of *L. litangensis* are monophyletic clade and then nested in *Ligusticopsis* genus (ML/BS ≥ 95, BI/PP ≥ 0.95). Although *L. litangensis* was related to *L. capillacea* with weak support in the ITS tree, *L. litangensis* can be discriminated from *L. capillacea* by its very short pedicels in the compound umbels (versus the long pedicels in compound umbels), oblong-globose mericarp (versus ovate) and a style that is significantly longer than petals (versus a style that is significantly shorter than petals). Similarly, although *L. litangensis* was related to *L. dielsiana* in the cpDNA tree, *L. litangensis* can be distinguished from *L. dielsiana* by its height of 5–10 cm (versus 20–50 cm), very short pedicels in compound umbels (versus long pedicels in compound umbels), subulate calyx teeth (versus linear-lanceolate), 1–2 vallecular vittae (versus 1–3) and 3–4 commissure vittae (versus 4–6).

Consequently, there is no doubt that *L. litangensis* is a new member of *Ligusticopsis* in terms of the morphological characteristics and phylogenetic evidence. Furthermore, our results support prior research identifying *Ligusticopsis* as an independent natural genus [6] and provide new insights for subsequent studies on the phylogenetic relationships and evolutionary processes of *Ligusticopsis*.

### 3.4. Taxonomic Treatment

*Ligusticopsis litangensis* R.M. Tian and S.D. Zhou sp. nov. (Figure 1 and Figure 2).

Diagnosis: *Ligusticopsis litangensis* can be identified by the following morphological features such as fibrous remnant sheaths covering the base of the stem, obviously developed calyx teeth, oblong-ovoid fruit dorsoventrally compressed, enlarged and winglike lateral ribs, extremely conspicuous single umbels, unequal length umbrellas and a small number of commissural vittae.

Type: CHINA. Sichuan: Litang County, in alpine grassland; 30°12′46.31′’N, 99°54′38.6′’E; elevation 4100 m a.s.l., 29 September 2022, TRM 2022092901 (holotype: SZ) (Figure S1).

Etymology: The species is named after Litang County, Sichuan Province, China, where it is the type locality.

Description: Perennial low stem grass, plants 5–15 cm. Roots long conical, 5–8 cm long, multi-branched, stem base densely covered with fibrous remnant sheaths. Leaves basal, petiole base expanded into sheaths; leaves’ blade outline oblong-lanceolate, 5–8 × 1–3 cm, 2-pinnate; pinnae 4–7 pairs, sessile, 2–3 × 0.6–1 cm, ultimate segments lanceolate. Very short pedicels in compound umbels; rays 10–15, unequal, glabrous, up to about 15 cm long. Bracts 2–4, 1-pinnate; bracteoles 12–15, 1-pinnate, white pubescent. Developed calyx teeth, linear to lanceolate. Petals white, obovate, apex reflexed; stylopodium conical, style reflexed. Fruit oblong-ovoid, dorsoventral compressed, dorsal and intermediate ribs keeled, winged lateral ribs; 1–2 vittae in each furrow, 4 on commissure; plane seed face.

Phenology: the flowering and fruiting period is from August to October.

Distribution, habitat: *Ligusticopsis litangensis* is distributed in Litang County, western Sichuan, China, and grows in the alpine meadow at an altitude of 4000–4500 m.

Key to the *Ligusticopsis* species:


1Plants densely villous or strigose2Plants nearly smooth42Rays of umbel almost draw from the base, extremely elongated
*L. hispida*
Plants compound umbels with long pedicels, umbrella not elongated33Bracts well developed, 2-pinnately divided
*L. involucratum*
Bracts caducous, 1-pinnately divided
*L. capillaceum*
4Umbels predominantly simple, rays drawn from base very unequal
*L. litangensis*
Compound umbels, rays subequal55Basal leaves and lower stem leaves are 1-pinnately compound
*L. integrifolia*
Basal leaves and lower stem leaves are 2–4-pinnately compound66Calyx teeth inconspicuous7Calyx teeth prominent87Bracteoles margin not membranous
*L. modesta*
Bracteoles with white membranous margin
*L. oliverianum*
8Stems unbranched, scapiform, cauline leaves usually absent
*L. scapiforme*
Stems simple or branched, cauline leaves present99Calyx teeth linear10Calyx teeth subulate1110Bracteoles with broad white membranous margins
*L. wallichiana*
Bracteoles without white membranous margins
*L. brachyloba*
11Leaf blade 1-2-pinnate, ultimate segments ovate to oblong-ovate
*L. rechingeriana*
Leaf blade 3-4-pinnate, ultimate segments linear
*L. dielsiana*



## 4. Materials and Methods

### 4.1. Plant Sample, Morphological Observation and Anatomy

The samples were collected in Litang County, Sichuan Province, China. The fresh green leaves were dried with silica gel and the mature fruits and inflorescences were preserved by the formaldehyde-acetic acid-ethanol method. The voucher specimens were stored in the Herbarium of Sichuan University (SZ) under the deposition number TRM2022092901. Based on the conservation of this new species and subsequent research, we collected 10 individuals with mature fruits and 3 individuals with inflorescences in the field and brought them back to the laboratory for relevant morphological observation and anatomical research and preservation as type specimens. Regarding the important fruit anatomy study, we selected three fruits in each individual plant for the investigation, in order to ensure the comprehensiveness and credibility of the results. In addition, all type specimens of closely related species on the website were consulted and carefully compared with the new species.

The morphological identification characteristics of the genus *Ligusticopsis*, which have a widely recognized practical value, have been described more clearly by Li et al. [5]. Therefore, we define the morphology of the new species and closely related species with reference to the criteria proposed by Li et al. [5] in combination with the type of specimens and the flora of China. The morphological features were observed with a Nikon SMZ25 stereoscopic microscope (Nikon, Tokyo, Japan) (Figure 2). The morphology of the roots, stems, leaves, inflorescences and bracts of this new species was observed directly under the stereomicroscope by photographing and recording the relevant features. The mature mericarps collected in the field were preserved in FAA fixative and used for subsequent experiments in the laboratory. The mericarps with well-preserved structures were selected, blotted with absorbent paper to absorb the excess FAA fluid, dried naturally and then photographed under the stereomicroscope to preserve their dorsal views. Our preliminary observations revealed that the furrowed vittae and commissure vittae of all mericarps were long. In the formal operation, vertical horizontal slices of the central part of the mericarps were made with a double-layer blade, and the cross-sectional slices were placed under a stereomicroscope for observation and photography. Ten mericarps with well-preserved structures were randomly selected and the number of furrowed vittae and commissural vittae was directly counted. All these features were compared with the taxon of genus *Ligusticopsis* (Table S1).

### 4.2. DNA Extraction, Sequencing, Assembly and Annotation

Total genomic DNA was extracted from silica gel dried leaves with a modified CTAB method [33]. The ITS sequence was amplified using the forward primers ITS4 (5’ -TCC TCC GCT TAT TGA TAT GC-3’) and reverse primers ITS5 (5’ -GGA AGT AAG TCG TAA ACA GG-3’) [34]. We operated a 30 µL amplification system, including 15 μL 2 × Taq MasterMix (CWBIO, Beijing, China), 10 µL ddH2O, 1.5 μL forward primer, 1.5 μL reverse primer, and 2 µL total DNA. The PCR reflected parameters of amplification were initial denaturation at 94 °C for 3 min, followed by denaturation at 94 °C for 45 s, annealing at 54 °C for 60 s, extension at 72 °C for 90 s, 30 cycles and finally extension at 72 °C for 10 min. All PCR products were separated on 1.5% (*w*/*v*) agarose TAE gels, and qualified PCR products were sent to Sangon Bioengineering Company (Sangon, Shanghai, China) for sequencing. In addition, 50 µL of extracted total DNA solution was sent to Novogene (Beijing, China) for total genomic DNA sequencing and library construction, with a sequencing depth of 5 G. The sequencing platform was Illumina Novaseq 6000 (Illumina, San Diego, CA, USA), and Nova-PE150 sequencing strategy was used for double-ended sequencing. The obtained clean data were assembled using NOVOPlasty v.2.7.1 [35] for plastids whole genome sequence. Seed selection of *L.involucratum* rbcL gene (GenBank accession No: NC049054). GENEIOUS R11 [36] was used to annotate the whole plastid genome, the seed sequence was used as the reference sequence and manual correction was performed. The PhyloSuite program was used to extract protein-coding sequences (CDS) from plastid genomes [37]. A physical map of the plastid genome of a new species was generated using OGDraw v1.3.1 [38].

Meanwhile, the newly sequenced ITS sequences and plastid genome data were submitted into the NCBI and the accession numbers were presented in Table S4.

### 4.3. Phylogenetic Analyses

To determine the phylogenetic position of *L.litangensis*, 41 ITS sequences and 40 protein-coding sequences (CDS) were utilized to reconstruct the phylogenetic tree (Table S4). Among them, *Bupleurum krylovianum* Schischk. ex Kryl. and *Bupleurum chinense* DC. were chosen as the outgroup according to previous studies [39]. Two datasets were aligned using MAFFT v7.221 [40] and then manually adjusted in MEGA7.0 [41] respectively. Maximum likelihood analysis and Bayesian inference were performed to build the tree. ML analysis was performed in RAxML v8.2.8 [42] software, and phylogenetic trees were constructed using the GTR + G + I model and 1000 bootstrap tests (BS) replicates. For the BI analyses, ModelFinder [43] was used to test the optimal models (GTR + G) for them, respectively. Bayesian inference (BI) was carried out in the MrBayes 3.2.7 [44] software, with running 1 × 107 generations of Markov Chain Monte Carlo (MCMC), sampling every 1000 generations and discarding the first 20% of the tree as burn-in. Results of phylogenetic analyses were visualized by the online tool iTOL [45].

### 4.4. Comparative Plastome Analyses

With the development of the second-generation sequencing technology, an increasing number of researchers are using the plastid genome for the phylogenetic and genomic comparative studies [46,47,48,49,50,51], helping to address some phylogenetic problems that cannot be resolved solely by molecular fragments.

The whole plastid genomes of twelve annotated *Ligusticopsis* species were uploaded onto the online program IRscope [52] for comparison. The boundary images of the LSC, SSC and IR regions of the whole plastid genome were mapped online, and then the final view of IR boundary was obtained by manual revision.

To determine whether specific patterns of structural variation existed in the 12 plastomes, a comparative visual analysis of gene arrangement was performed using the Mauve comparison program [53], which was set by default in Geneious v9.0.2 [36].

The online program mVISTA [54] was used to analyze the sequence diversity of the plastid genome sequences of these twelve *Ligusticopsis* species. The parameters were set according to the Shuffle-LAGAN model, and the model species *L. rechingerana* was used as the reference.

### 4.5. Codon Usage and SSRs Analyses

The coding protein sequence (CDS) was screened from 12 plastomes of *Ligusticopsis* using Geneious v9.0.2 [36]. After removing the CDS with bases less than 300 bp and duplicates, a total of 53 CDS were selected. Then, the 53 CDS were connected end to end and analyzed for codon bias for each species of *Ligusticopsis* using the CodonW v1.4.2 program [55]. Finally, a heatmap was drawn using TBtools [56].

Simple sequence repeats are widely distributed in the genomes of higher organisms [57]. In our research, the MISA software [58] was used to identify simple repeated sequences in the whole genome of 12 species of *Ligusticopsis*. The corresponding parameters are set as follows: the minimum repetition of the mononucleotide is 10, the minimum repetition of dinucleotides is 5, the minimum number of repeats of trinucleotides is 4, and the minimum number of repeats of tetranucleotides, pentanucleotide and hexanucleotides are all 3.

## 5. Conclusions

The complete plastomes of *L. litangensis* were sequenced, assembled and annotated in our research. Based on comparative plastomes analysis, we concluded that the plastomes of 12 *Ligusticopsis* species, including the new species, are highly conserved in terms of genome structure, gene content and type, number and type of SSRs and codon usage bias. Significantly, the tree topologies obtained from ML and BI analyses based on the ITS data and plastome data firmly supported that three individuals of *L. litangensis* are monophyletic clade and then nested in the *Ligusticopsis* genus (ML/BS ≥ 95, BI/PP ≥ 0.95). Furthermore, the morphological characteristics of *L. litangensis*, such as the root neck densely covered with fibrous withered leaf sheaths, pinnate bracts, developed calyx teeth, strongly compressed mericarps, keeled dorsal and middle ribs, winged lateral ribs, 1–2 vittae per furrow and 3–4 vittae on the commissure. In conclusion, our plastid phylogenomic and morphological evidence robustly supports that *L. litangensis* is a new member of *Ligusticopsis*, and the results of our research have substantial implications for the phylogeny, taxonomy and evolution of the *Ligusticopsis* genus.

## Figures and Tables

**Table 1:** Comparison of genome content of 12 *Ligusticopsis* species plastomes. Pseudogenes not included.

Species	LSC Length (bp)	SSC Length (bp)	IR Length (bp)	Total Genome		Number of Genes			
				Length (bp)	GC (%)	Total	CDS	rRNA	tRNA
*L. litangensis*	91,559	17,669	19,127	147,482	37.4	129	85	8	36
*L. dielsiana*	91,666	17,582	19,415	148,078	37.4	129	85	8	36
*L. capillacea*	91,907	17,503	19,199	147,808	37.5	129	85	8	36
*L. scapiformis*	92,214	17,581	19,156	148,107	37.5	129	85	8	36
*L. rechingerana*	91,813	17,654	19,529	148,525	37.3	129	85	8	36
*L. brachyloba*	92,265	17,588	19,390	148,633	37.4	129	85	8	36
*L. hispida*	91,846	17,627	19,162	147,797	37.4	129	85	8	36
*L. integrifolia*	92,305	17,575	19,158	148,196	37.5	129	85	8	36
*L. involucrata*	91,782	17,560	19,205	147,752	37.4	129	85	8	36
*L. modesta*	92,247	17,568	19,159	148,133	37.5	129	85	8	36
*L. wallichiana*	92,281	17,567	19,373	148,594	37.4	129	85	8	36
*L. oliveriana*	92,262	17,558	19,279	148,378	37.5	129	85	8	36
*L. litangensis*	91,559	17,669	19,127	147,482	37.4	129	85	8	36

## Data Availability

The newly yielded ITS sequence of *L. litangensis* and *L.oliveriana* are submitted to the NCBI with accession numbers OP902933, OP902934, OP902935 and OP882296, respectively. The newly yielded plastome of *L. litangensis* are submitted to the NCBI with accession numbers OP899836, OP899837 and OP899838.

## Supplementary Materials

The supporting information can be downloaded at: https://www.mdpi.com/article/10.3390/ijms24087419/s1.
