# Supplementary material for: Plastid Phylogenomic Analyses Reveal a Cryptic Species of Ligusticopsis (Apiaceae, Angiosperms)
Source: Int J Mol Sci. 2023 Apr 18;24(8):7419. doi: 10.3390/ijms24087419 (PMC10138589; doi:10.3390/ijms24087419)
Supplement: Supplementary file 1 [file ijms-24-07419-s001.zip › Figure S1.pdf]

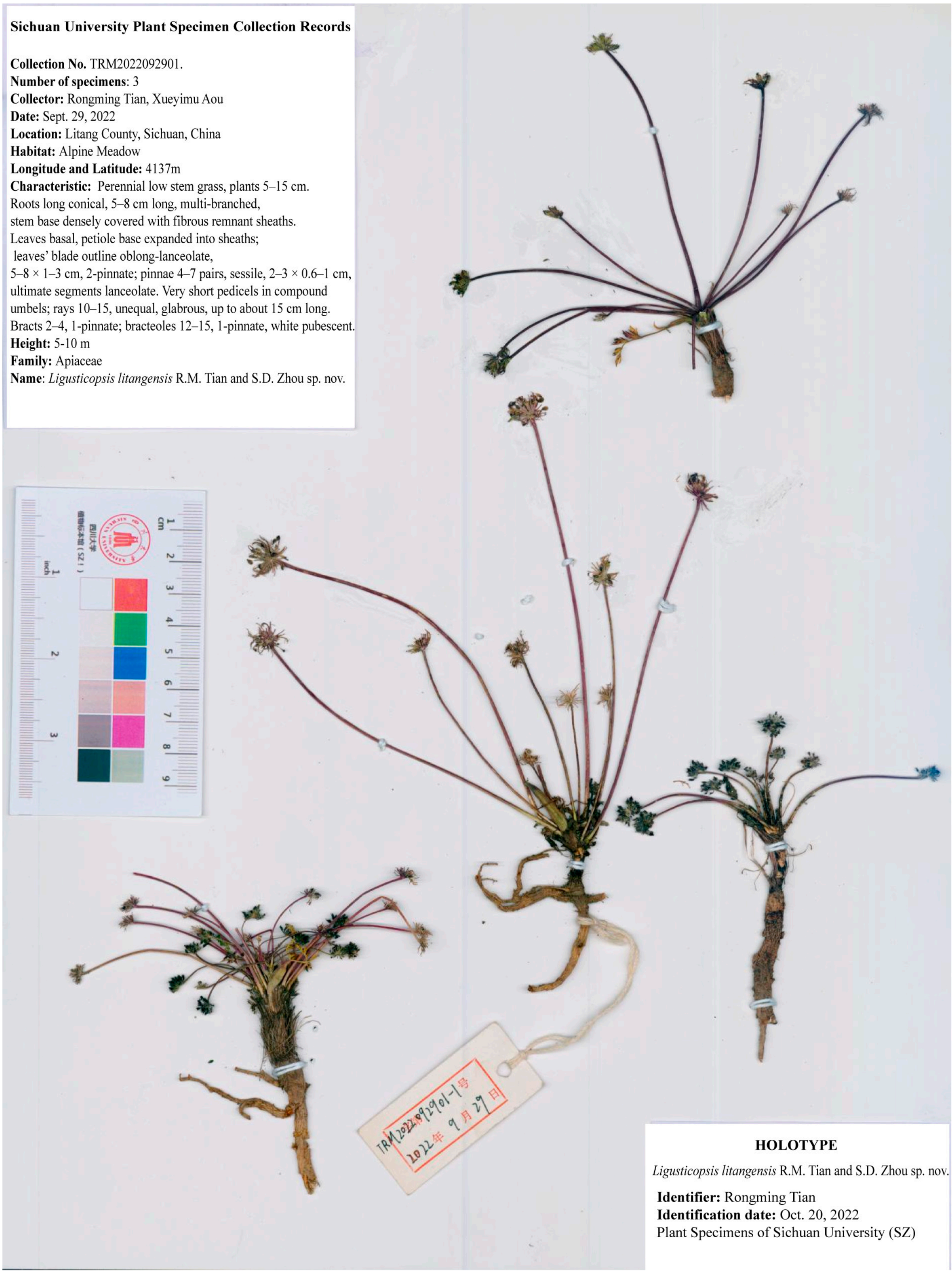

Figure S1. Holotype of *Ligusticopsis litangensis*. Table A4: Voucher details and GenBank accession numbers of taxa included in this study.
